# Transdermal and Topical Drug Administration in the Treatment of Pain

**DOI:** 10.3390/molecules23030681

**Published:** 2018-03-17

**Authors:** Wojciech Leppert, Malgorzata Malec–Milewska, Renata Zajaczkowska, Jerzy Wordliczek

**Affiliations:** 1 Department of Palliative Medicine, Poznan University of Medical Sciences, Osiedle Rusa 55, 61–645 Poznan, Poland; 2 Department of Anesthesiology and Intensive Care, Medical Centre of Postgraduate Education, 01-813 Warsaw, Poland; lmilewski@post.home.pl; 3 Department of Interdisciplinary Intensive Care, Jagiellonian University Medical College, 31-008 Krakow, Poland; renia356@poczta.onet.pl (R.Z.); mswordli@cyf-kr.edu.pl (J.W.); 4 Department of Anesthesiology and Intensive Therapy, University Hospital, 31-501 Krakow, Poland

**Keywords:** adverse effects, analgesics, pain, topical drugs, transdermal opioids

## Abstract

The comprehensive treatment of pain is multidimodal, with pharmacotherapy playing a key role. An effective therapy for pain depends on the intensity and type of pain, the patients’ age, comorbidities, and appropriate choice of analgesic, its dose and route of administration. This review is aimed at presenting current knowledge on analgesics administered by transdermal and topical routes for physicians, nurses, pharmacists, and other health care professionals dealing with patients suffering from pain. Analgesics administered transdermally or topically act through different mechanisms. Opioids administered transdermally are absorbed into vessels located in subcutaneous tissue and, subsequently, are conveyed in the blood to opioid receptors localized in the central and peripheral nervous system. Non–steroidal anti–inflammatory drugs (NSAIDs) applied topically render analgesia mainly through a high concentration in the structures of the joint and a provision of local anti–inflammatory effects. Topically administered drugs such as lidocaine and capsaicin in patches, capsaicin in cream, EMLA cream, and creams containing antidepressants (i.e., doxepin, amitriptyline) act mainly locally in tissues through receptors and/or ion channels. Transdermal and topical routes offer some advantages over systemic analgesic administration. Analgesics administered topically have a much better profile for adverse effects as they relieve local pain with minimal systemic effects. The transdermal route apart from the above-mentioned advantages and provision of long period of analgesia may be more convenient, especially for patients who are unable to take drugs orally. Topically and transdermally administered opioids are characterised by a lower risk of addiction compared to oral and parenteral routes.

## 1. Introduction

Pain is defined as a subjective phenomenon that is associated with actual or potential damage to tissues. Typically, two components are found: sensory, associated with information of noxious stimuli, and emotional, which is associated with a patient reaction to painful stimuli that is often associated with psychological reactions and an individual sensitivity to pain. There are distinct types of pain regarding its time frame and pathophysiology. With respect to the former, pain may be divided into acute, which typically lasts up to three months, and chronic, which is present for a longer period of time. Regarding pathophysiology, pain may be divided into nociceptive (somatic, visceral), which is associated with damage of different internal organs, musculoskeletal system, soft tissues and the skin with intact nervous systems, and neuropathic pain, which is associated with damage or disease of the somatosensory nervous system, regardless of possible causes. It is common that mixed (both nociceptive and neuropathic) pain syndromes are present, for example in cancer patients with bone metastases.

Patients diagnosed with chronic pain usually need a holistic approach to their numerous needs and typically require management that is comprised of pharmacotherapy, along with non–pharmacological measures. Regarding pharmacology, several groups of analgesics are available, and the general rule is to combine drugs with different modes of analgesic action, which allows for improved analgesia and reduced adverse effects. Typically, one or more drugs from three groups of analgesics are administered: non–opioids, opioid analgesics, and adjuvant analgesics. Apart from the antinociceptive efficacy and adverse effects profile, the route of analgesic administration plays a crucial role in achieving effective analgesia, as well as in minimizing adverse events.

**Figure 1:**
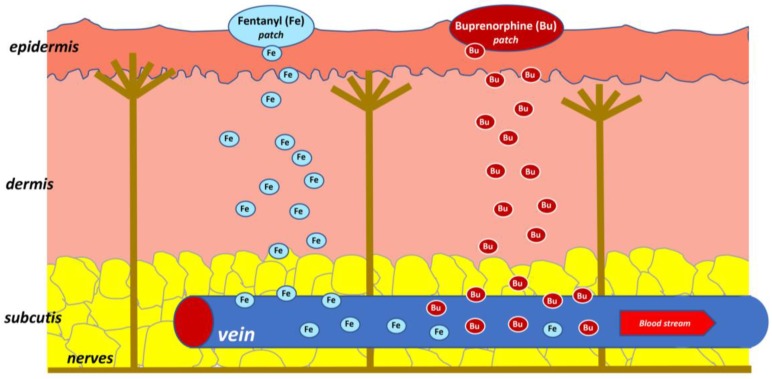
Transdermal application for systemically acting drugs.

**Figure 2:**
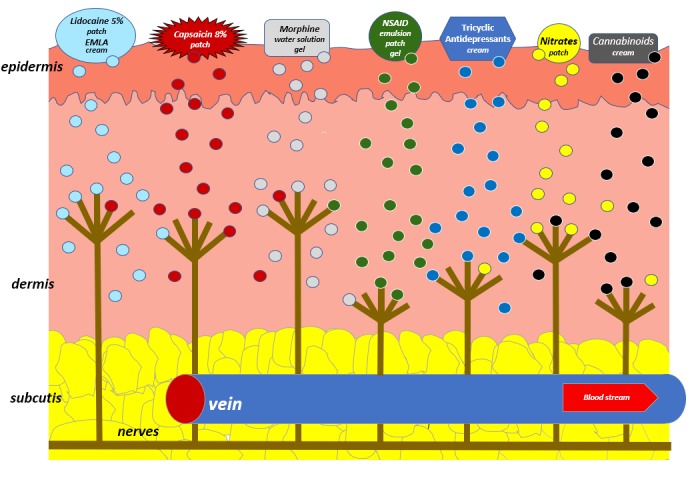
Topical application for locally acting drugs.

A drug administration route should be carefully selected while strongly taking into account the clinical status of patients, comorbidities, and age [1]. An important factor in choosing an optimal route of analgesic administration is the acceptance of patients. There are several routes of analgesics administration: oral, sublingual, buccal, intranasal, inhaled, subcutaneous, intravenous, intramuscular, rectal, intramedullary, intrathecal, transdermal and topical. In this article, transdermal and topical routes of analgesics are presented, which, apart from a non–invasive method of administration, may provide some more advantages such as high efficacy along with a beneficial profile of adverse effects. This benefit especially refers to topical routes, as this usually offers avoidance of potentially serious adverse effects when analgesics are administered by systemic routes. For example, opioids administered by a transdermal route are absorbed into vessels located in subcutaneous tissue and, subsequently, are conveyed in the blood to opioid receptors localized in the central nervous system (CNS) (Figure 1) [2]. Non–steroidal anti–inflammatory drugs (NSAIDs) applied topically are absorbed into systemic circulation in a limited percentage, and the main mechanism of action is based on a high concentration in the structures of joints and the provision of local anti–inflammatory effects. Lidocaine and capsaicin in patches, capsaicin in cream, EMLA cream, and creams containing antidepressants (e.g., doxepin, amitriptyline) act mainly locally in tissues through receptors and/or ion channels (Figure 2). Transdermal and topical routes of opioid administration are also associated with a lower risk of addiction compared to oral and parenteral routes of opioid analgesics administration.

## 2. Opioids Administered Transdermally

Benefits associated with the administration of opioid analgesics through a transdermal route are as follows:

- The use of the transdermal route allows for the avoidance of problems associated with a disturbed absorption of a drug from the gastrointestinal (GI) tract or other GI problems (i.e., swallowing difficulties, nausea, vomiting), as well as the elimination of the first pass effect through the liver.
- Low concentrations of drugs and small fluctuations of their concentration in the blood serum guarantee long–lasting analgesia with a lower number of adverse effects, especially nausea, vomiting, and constipation.
- The transdermal form of drugs provides simplicity and convenience of administration, increases compliance, and improves patients’ quality of life.

Features that should be fulfilled by a transdermal opioid are as follows: small molecular weight, high lipophilicity, high efficacy to compensate for limited absorption, low melting temperature, relatively short half–life, low daily dose, system dosing providing absorption from a relatively small area, and matrix patches in which a total amount of a drug is localized homogenously in an adhesion layer. The above technology ensures regulation of the release of an opioid based on a gradient concentration between a patch and the skin. The damage of a patch does not evoke an uncontrolled release of an active substance and enables the division of a patch into smaller parts in order to administer a lower dose of a drug, which is especially relevant in older patients. Natural features listed above ease the crossing of a drug through the skin and are possessed by two opioid analgesics: fentanyl and buprenorphine (Table 1).

### 2.1. Fentanyl

The first drug used in the treatment of pain through the transdermal route was fentanyl. Patches of fentanyl release 12.5, 25, 50, 75, and 100 µg/h (Table 2). A patch should be applied in the region of intact, non–irradiated skin, on flat parts of the thoracic cage and/or upper arm.

In patients with fever and in those with excessive sweat, the absorption of the drug may be disturbed due to a layer of sweat separating the active surface of a patch from the skin. Patients with a patch may take a bath, shower, and swim, although they should not use solariums and saunas.

After the fentanyl patch is first applied, the beginning of its analgesic action is delayed by up to approximately 12 h. Therefore, for this period of time, patients should be treated with other analgesics. In most patients, the fentanyl analgesic effects persist for 72 h (3 days) and, for this period of time, a patch should be administered. In some patients, a decrease of the analgesic effect is observed on the third day. In these patients, a change of patch every 48 h (2 days) may be considered. A fentanyl dose proportion to 90% undergoes biotransformation in the liver through the cytochrome P–450 enzyme CYP3A4, forming an inactive metabolite, norfentanyl. The rest of the drug (10%) is not metabolized. Fentanyl and its metabolite are excreted by the kidneys. The advantages of fentanyl are as follows: compared to morphine, it is less constipating, emetogenic, sedative, and does not release histamine; it is relatively safe in patients with renal failure.

Fentanyl in patches is not recommended for the treatment of acute postoperative pain due to a slow onset of analgesic action after the first patch application. However, in postoperative analgesia, it is possible to use fentanyl administered by a transdermal route through a special micropump system (IONSYS), in which the acceleration of opioid absorption is achieved through an iontophoresis process. In this system, a small micro–pump with a diameter of an index finger is attached to the arm skin. After pushing a special button, a dose of fentanyl equaling 40 µg is applied. For safety reasons, the device possesses a programmed 10–minute refraction period when administration of the drug is impossible. The number of possible doses administered per day (24 h) is 80. In patients treated with fentanyl pumps, a constant monitoring of the respiratory system function is required. Fentanyl products administered by transmucosal routes (i.e., intranasal, buccal, and sublingual) are recommended for the management of breakthrough (episodic) pain [3].

### 2.2. Buprenorphine

Buprenorphine is available in patches releasing 35, 52.5, and 70 µg/h. The maximum recommended dose of buprenorphine in Europe is 140 µg/h. In older patients, a starting dose may equal 8.75 µg/h or, more frequently, 17.5 µg/h (a fourth and half of a patch, respectively). A change in the dose should take place after at least two consecutive applications of a patch. The pharmacokinetic steady–state and an equilibrium drug distribution between blood and cerebrospinal fluid are reached after at least five plasma half–lives. Rules of patch applications are the same as that for fentanyl. To decrease the risk of dermatitis induced by a buprenorphine patch, some clinicians recommend keeping the patch in the air to evaporate gaseous substances contained in a patch before putting the patch on the skin; this is recommended only for an original buprenorphine product. Original buprenorphine patches are normally changed every 3.5 or 4 days. Dosing twice a week is more convenient for many patients because of fixed days for patch changes. A generic product is normally changed every three days. In Europe, a seven–day buprenorphine patch is available releasing 5, 10, and 20 µg/h (Table 3).

Buprenorphine has been used in clinical practice for over 30 years, but the interest in the drug increased after introducing transdermal products. Buprenorphine is a semi–synthetic derivative of thebaine. The buprenorphine molecule contains a basic skeleton of morphine, but there are significant differences between their structures. It may be predicted that buprenorphine displays some unique and distinguished pharmacological and clinical features while preserving general morphine properties.

Buprenorphine strongly binds to opioid receptors, displaying the highest affinity compared to other commonly used opioids. Buprenorphine binds to opioid receptors more slowly and dissociates more slowly than fentanyl, which is associated with less risk of developing opioid withdrawal. Despite the fact that buprenorphine in therapeutic doses displays a high affinity with opioid receptors, its degree of binding with opioid receptors is small (less than 50%). This leaves a significant percentage of opioid receptors free, making it possible to use buprenorphine concurrently with other opioids. However, concurrent treatment with other opioids renders only additive effects. Buprenorphine is a partial agonist of μ opioid receptors. Therefore, a curve dose/response is not a simple line but an “S” shape. In the range of therapeutic doses, the curve is linear for doses below 7 mg/day, i.e., each dose increment induces an improvement in analgesia. This means that, in this dose range, the drug acts as a pure opioid agonist. A ceiling effect of analgesia is achieved when daily doses exceed 16 mg, which are not used in the clinical practice of pain management. A potentially important, although not fully recognized, role of buprenorphine is the interaction with orphanin receptors, ORL–1. This receptor is similar to opioid receptors and binds nociceptin, which, after the activation of these receptors, antagonizes the analgesic effects of opioids.

After absorption from a transdermal patch, buprenorphine binds to blood proteins in circulation to a large extent (96%). This may affect its pharmacokinetics and action, especially in patients with cachexia. In the liver, the drug is metabolized through the cytochrome P–450 enzyme CYP3A4 to become an active metabolite, norbuprenorphine. A third of the dose administered by a transdermal route is excreted via the kidneys and two–thirds of the drug is excreted with the stool. Transdermal buprenorphine is recommended for cancer and non–cancer patients with moderate to severe pain intensity of musculoskeletal, neuropathic, and visceral pain. Apart from pain management, buprenorphine is used in substitution therapy for patients diagnosed with drug addiction. For this indication, its effectiveness is comparable with methadone.

Drug concentrations in the blood serum that can render analgesia are achieved within 12–24 h after a first patch application, which accounts for the fact that buprenorphine is similar to transdermal fentanyl and should not be used in the treatment of patients with acute and breakthrough (episodic) pain. Transdermal buprenorphine renders a stable concentration of the drug in the blood serum, similar to transdermal fentanyl. It was demonstrated that the profile of adverse effects of transdermal buprenorphine is similar to other opioids. Long–term treatment with the drug is characterized by a low frequency of constipation and CNS adverse effects, such as nausea, vertigo, and weakness. The low frequency of adverse effects on the CNS might be due to the antagonist action of buprenorphine on the kappa opioid receptor (KOR). The low frequency of adverse effects in the GI tract is probably associated with a high lipophilicity of the drug. The safety profile of buprenorphine is better compared to other opioids.

Buprenorphine displays less neurotoxic effects compared to other opioids (such as fentanyl), especially in older patients and those with dementia. Clinical studies have demonstrated that buprenorphine dose increments allowed the achievement of good analgesia with a limited risk of respiratory depression. This differentiates buprenorphine from other “strong” opioids that display dose dependent respiratory depressant effects. There is evidence from animal studies that most opioids (apart from buprenorphine and oxycodone) induce some degree of suppression of the immune system through modification of NK (natural killer) cells function, lymphocytes T, action of interleukin–2, or interferon gamma. Lack of negative impact on the immune system may improve safety of the drug regarding infections and cancer dissemination. Moreover, a significant advantage of buprenorphine, which is similar to fentanyl, is high safety in using the drug in patients with renal failure [4].

Tolerance development for analgesia during treatment with transdermal buprenorphine was explored in comparative studies with transdermal fentanyl. The increase in demand for fentanyl was at 2% per day and at 0.1% per day for buprenorphine, which is a result of the broader mode of action of buprenorphine on opioid receptors. Because of the strong analgesic potency of transdermal buprenorphine and the low frequency of tolerance development for analgesia, high doses of the drug in clinical practice are rarely required. The most frequently used dose range is 35–70 µg/h.

## 3. Opioids Used Topically

Opioids induce analgesia by binding to opioid receptors in the CNS (spinal cord and brain). Opioid receptors have also been shown to be present on peripheral sensory nerve terminals in inflamed tissues. Clinical studies have demonstrated analgesic efficacy when opioids are applied topically in certain situations such as skin ulcers and oral mucositis. The advantage of administering an opioid topically is the avoidance of systemic adverse effects such as nausea, constipation, and sedation.

Morphine may also be administered through the mucosa. A 0.1–0.2% morphine–water solution may be used topically in the treatment of pain associated with changes at the oral mucosa evoked by radiotherapy and chemotherapy (i.e., rinsing mouth with morphine aqueous solution). A 0.1% morphine gel is applied on skin ulcerations, skin damage induced by radiotherapy, and pressure sores [5]. Before an application of these morphine formulations on the skin surface, a surgical elaboration of a wound should be conducted because topical opioids may hamper wound closure due to the inhibition of peripheral neuropeptide release into the healing wound. The duration of action of topical morphine has been reported to be as long as 7–12 h, which is longer compared to morphine administered by oral or parenteral routes (4–6 h). There are ongoing studies on the use of morphine administered systemically in transdermal patches [6].

## 4. Analgesics Administered Topically

### 4.1. Nonsteroidal Anti–Inflammatory Drugs

All joint structures, apart from cartilage, are very rich in nerves. Thus, pain in joints and bones are usually of severe intensity. Prostaglandins play a main role in the process of inducing peripheral inflammatory pain; therefore, NSAIDs, which have a basic mechanism of action of blocking prostaglandin formation, are an important component in the treatment of this type of pain. The concentration of an NSAID in the blood serum determines its analgesic effects, and its concentration in a joint decides its anti–inflammatory effect.

NSAIDs administered systemically achieve a high concentration in the blood serum and may induce adverse effects in the circulatory system, GI tract, kidneys, and liver [7]. In contrast to systemic administration, a local application of NSAIDs supports analgesic and anti–inflammatory effects with little risk of aforementioned, potentially serious adverse effects. NSAIDs administered topically are normally used for a period of one to two weeks and are effective in the following types of pain: musculoskeletal, mainly after injuries, soft tissue pain and rheumatic diseases (efficacy of 18–92%).

Numerous studies have proven that, after local NSAID administration on the skin, the concentration of a drug in the blood serum reaches only 5–15% of the concentration achieved after systemic administration, whereas some parts of a drug penetrates to the synovial fluid and synovial membrane. Penetration of drugs may be significantly improved through the use of ultrasound and iontophoresis. Concentration of NSAIDs after topical administration in the joint cartilage and in the meniscus is 4–7 times higher compared to that after an oral administration of NSAID; in tendinous cots and in the bursa, it is a few dozen times higher compared to that after an oral administration of NSAID. The NNT (the number needed to treat) for topically administered NSAIDs is 3.9 for acute pain syndromes and 3.1 for chronic pain. In chronic pain with an accompanying inflammatory process, combining a local and orally administered NSAID seems to be a good approach (Table 4).

### 4.2. Local Anesthetics

#### 4.2.1. EMLA Cream

EMLA cream is an oil–water lotion containing lidocaine and prilocaine in a 1:1 ratio. It is a unique local anesthetic formulation, which, thanks to its large water content, is easily absorbed through the skin. In addition, due to the fact that the active substance is present in the form of more active alkaline compounds contained in small lipid drops suspended in water and not in a ionized salt form, it is possible to obtain an effective skin anesthesia up to approximately 0.5 cm in depth. This product is a eutectic mixture, which means that the composition of the drug is matched in a way that the melting point of the mixture of both components is different than the melting points of the individual components. In the temperature of human skin, this product preserves a liquid form, thanks to which it has a chance of being absorbed through the skin and inducing its anesthesia. According to the literature, this product may be applied on quite a large skin surface, even over 400 cm^2^, in a single dose of 30–50 g. A gram of cream contains 25 mg lidocaine and 25 mg prilocaine.

Indications for EMLA cream administration include the following: skin anesthesia alleviating acute pain in surgery, limited to small depth of the skin (i.e., excision of minor skin changes, fixing intravenous lines, painful biopsies) and localized peripheral neuropathic pain, for example postherpetic neuralgia (PHN), especially in young patients with contraindications to sympathetic system blocks. When using EMLA cream on a large surface, it is necessary to monitor circulatory system function, lidocaine, prilocaine, and methemoglobin concentrations.

#### 4.2.2. Patches Containing 5% Lidocaine

Distinguished from EMLA cream, patches containing 5% lidocaine act mainly locally through a pathology-dependent form voltage sodium channels (or voltage gated sodium channels, VGSC) formed in an injured nerve. VGSC gather in places of nerve injury, initiating repetitive ectopic excitations. These channels mark a high ability of lidocaine binding and a slow dissociation. Lidocaine released from a patch penetrates through the skin and, to a negligible degree, is absorbed into vessels; therefore, it does not induce circulatory complications and there is no need to monitor circulatory system function. Lidocaine contained in a patch binds with internal wall VGSC, which are formed in nerve endings and keratinocytes. As a result of blocking VGSC, ectopic excitations are inhibited, but there is no blocking of afferent nerve conduction (i.e., lack of numbness of the skin).

The second mechanism of lidocaine is associated with an inhibition of the release of nociception process mediators by keratinocytes. Keratinocytes participate in a system of signal transfer, and their activation induces sensitization and depolarization of primary sensory nerve endings through purinergic receptors (P2X) and an increase in expression of neurokinin receptor NK1 activated by substance P release, which leads to the activation of primary nociceptive sensory nerve endings. Lidocaine in patches additionally induces an effect of skin cooling (hydrogel bandage) and provides mechanical protection of skin areas involved in the disease process.

Recommendations for the use of 5% lidocaine include the following: as a first line drug in the treatment of localized, peripheral neuropathic pain (especially with accompanying allodynia), alone or in combination with another first line drug and for central pain induced by spinal cord compression caused by a metastatic tumor to the subarachnoid space. The NNT for lidocaine in PHN is 4.4 and NNH (the number needed to harm) is approximately 28. The use of 5% lidocaine is effective in the following pain conditions: postherpetic neuralgia, painful diabetic neuropathy; its effectiveness is comparable to amitriptyline, capsaicin, gabapentin, and pregabalin, intercostal neuralgia, persistent postoperative pain (after thoracotomy, mastectomy, inguinal hernia surgery, amputation), meralgia paraesthetica, low back pain and muscle–fascial pain. The rules of patch application and adverse effects are as follows:

- Patches are applied on the skin in painful places once a day and left for 12 h.
- There should also be a break lasting 12 h, as a break in patch application minimizes the risk of local skin reactions.
- With one application, a maximum of three patches is allowed.
- The most frequent adverse effect of 5% lidocaine is a local skin irritation [8].

#### 4.2.3. 2% Lidocaine Gel

The drug displays good absorption from the mucosal surface and, through this, provides the possibility of superficial anesthesia. In chronic pain treatment, it is used, for example, in pain syndromes localized in the pelvis in women (i.e., perineal, intractable pain after sex).

### 4.3. Drugs Acting through Vanilloid Receptors TRPV1

#### Capsaicin

Capsaicin is a highly selective agonist of the vanilloid receptor from a group of transient receptors of potential TRPV1 (i.e., transient receptor potential cation channel subfamily V member 1 vanilloid receptor). Initially, capsaicin activates nociceptors in the skin, which evokes irritation and erythema induced by a release of vasoactive neuropeptides (substance P). After exposure to capsaicin, nociceptors in the skin are less sensitive to different stimuli; therefore, the late action of capsaicin is depicted as anesthesia.

After one week from the patch application containing capsaicin with a high (8%) concentration, the channels for calcium ions for the skin open, leading to the sharp influx of calcium to cells and subsequent reversible damage of mitochondria. This consequently induces long-term atrophy of peripheral skin nerves. Capsaicin–induced changes in nociceptors of the skin and in peripheral nerves are reversible. A return to the normal function of receptors and repair of nerve fibers are observed after 12 weeks. This mechanism of action of capsaicin patches with a high concentration should be classified as a neuro–destructive action [9].

The mechanism of action of capsaicin is also based on the elimination of the neurotransmitter substance P from nerve fibre endings. As a consequence, there is a reversible decrease in stocks of substance P and a decrease of pain transmission from peripheral nerve fibres to the CNS [10]. Capsaicin used in the form of ointment with a concentration below 1% is effective in pain syndromes such as syndrome of burning mouth, neuropathic face pain, trigeminal neuralgia, reflex sympathetic dystrophy, neuropathy in the course of HIV, osteoarthrosis, Guillain–Barré syndrome, fibromyalgia, and persistent postoperative pain (post–mastectomy pain syndrome). The use of capsaicin in the form of ointment requires numerous applications and is associated with persisting pain as a result of contamination with the dressings or beddings of a patient. Since 2009, 8% capsaicin has been available in the form of patches.

In clinical studies, a patch with 8% capsaicin demonstrated high efficacy in patients with postherpetic neuralgia (PHN) and neuropathy during the course of HIV, as well as in the treatment of neuropathy after chemotherapy. Studies are ongoing on the use of patches containing 8% capsaicin in patients with diabetic neuropathy; the high efficacy of capsaicin ointments with low concentration for this disease might suggest that a high concentration of capsaicin should be equally or more effective. A patch with capsaicin has dimensions of 14 × 20 cm (280 cm^2^) and contains 179 mg of capsaicin (640 µg capsaicin/cm^2^). A maximum of four patches are recommended. A patient must be informed that reactions, such as pain or burning, erythema, itch, and edema, may appear in the area where the patch is applied [11]. The adverse effects or disadvantages of capsaicin are as follows:

- Transient burning pain, erythema, and itch in the area of patch application.
- Adverse reactions are transient, self–limiting, and usually of mild or moderate intensity.
- No neurologic function limitations were found, apart from periodic disturbed hot sensation in the area of patch application.
- High price of the drug.
- The lengthy time duration of a procedure (approximately 3 h).

### 4.4. Tricyclic Antidepressants

The predominant mode of action of tricyclic antidepressants that are used in the treatment of neuropathic pain is the activation of descending pain inhibition through inhibition of the reuptake of serotonin and noradrenaline from the synaptic cleft. An additional consequence of this is potentiating opioid analgesia. Other tricyclic antidepressant modes of action comprise a blockade of NMDA (*N*–methyl–d–aspartate) receptors, adenosine receptors, and sodium channels. The latter mechanism of action (i.e., sodium channel blocking) might be a theoretical base for the beneficial action of this group of drugs in peripheral neuropathic pain, administered locally in the form of ointments or creams. Clinical studies demonstrate the efficacy of tricyclic antidepressants, especially doxepin and amitriptyline, in the treatment of peripheral neuropathic pain. Indications for a local administration of tricyclic antidepressants are the following pain syndromes: carpal tunnel syndrome, neuralgia of intercostal nerves, postherpetic neuralgia, complex regional pain syndrome, painful diabetic neuropathy, meralgia paraesthetica, coccydynia, costochondritis, mucositis after chemotherapy and intractable itch that accompanies, for example, atopic dermatitis.

In the case of an itch, an analgesic effect of tricyclic antidepressants is attributed to an antihistaminic effect. In spite of the proven efficacy of orally administered tricyclic antidepressants in the treatment of neuropathic pain, adverse effects in nearly one-third of treated patients often preclude use of these drugs. Therefore, particularly in the case of intolerance when tricyclics are administered by an oral route, local application is recommended [12].

#### 4.4.1. Doxepin

The active substance of the cream (Xepin) is 5% doxepin hydrochloride. The drug should be contained in temperatures below 27 °C. It should be applied as a thin layer on the skin surface, not exceeding 10%, three to four times a day, with a break of at least 3 to 4 h between applications. It is not recommended to use the cream under an occlusive bandage due to the risk of excessive absorption into the circulation. The recommended period of therapy in the case of an itch is eight days; in the treatment of peripheral neuropathic pain, treatment time is prolonged to four weeks. After application of the drug on the skin, hands should be accurately washed and contact of the drug with mucosa and the conjunctiva of the eye should be avoided.

The most frequent adverse effects are comprised of burning, tingling, or feeling a stinging sensation in the area of application. Other adverse effects induced by excessive absorption of the drug into the circulation are as follows: drowsiness, dizziness and headache, dry mouth, dry conjunctiva in eyes, blurred vision, and taste change. Very rarely, GI symptoms were reported such as nausea, vomiting, dyspepsia, diarrhea, or constipation. There were also symptoms such as ringing in ears, agitation, disorientation, urine retention, cardiac arrhythmia, changes in blood glucose levels, and fever. Caution is recommended when administering the drug in patients diagnosed with glaucoma, prostate hypertrophy, depression, severe heart, and liver disease. The drug is not recommended in children under the age of 12. No studies were conducted on the safety of the drug in pregnancy and during breast feeding. The most frequent adverse effects, which do not require treatment cessation, are discomfort (i.e., burning, itch, tingling, and stinging) in the region where the drug is applied on the skin. Drowsiness suggests the systemic absorption of doxepin [13]. More studies are required assessing the absorption of the drug after transdermal administration.

#### 4.4.2. 10% Amitriptyline

In a rat model of neuropathic pain, amitriptyline (ointment) administered locally on the injured paw effectively decreased pain intensity. This analgesic effect was reversed by systemic administration of caffeine, which is an antagonist of the adenosine receptor. It is suggested that at least part of the peripheral analgesic effect of amitriptyline is associated with peripheral adenosine receptors. The application of amitriptyline on the skin of a healthy patient did not induce an analgesic effect, which suggests that, in this case, a local action of the drug was predominant. There is slight evidence of the efficacy of tricyclic antidepressants in humans. Studies refer to a small number of patients, many of which have a relatively short follow-up period. This hinders the interpretation of the results.

### 4.5. Other Drug Groups Administered Locally

#### 4.5.1. Nitrates

Nitrates are mainly used in the treatment of ischemic heart disease. However, drugs forming this group also display analgesic and anti–inflammatory effects. It is known that exogenous nitrates stimulate the release of nitrogen oxide (NO) from endothelium cells. This substance is a very strong mediator in many systems, especially in the central and peripheral nervous systems. It seems that NO exerts its action through the stimulation of an increase in the concentration of guanylyl cyclase, increasing the level of cGMP. NO may also activate sensitivity to ATP potassium channels and, in this way, activate pain relief in the periphery. It was demonstrated that ointments or patches containing glycerol trinitrate (nitroglycerine) display analgesic efficacy in the following conditions: rheumatoid arthritis, ankylosing spondylitis, costochondritis, muscle–fascial pain, complex regional pain syndrome, bursitis, coccydynia, bone metastases, vulvodynia and venous leg ulcers.

There is data indicating that the local action of nitrates potentiates analgesic effects of “strong” opioids, which indicates that this effect is a result of nitrate absorption and because of their central mode of action. The use of nitrates may be especially useful in patients in whom, due to renal, liver, heart, and GI tract diseases, NSAIDs are contraindicated. The main adverse effect associated with a local application of nitrates is frequent headaches [14].

#### 4.5.2. α–2–Agonists (Clonidine)

Clonidine administered in transdermal patches effectively eases pain in the following diseases: migraine, face pain and complex regional pain syndrome. This route of clonidine administration is associated with typical systemic adverse effects such as hypotension, sedation, and dry mouth. This is typical for systemic administration. Topical administration of clonidine effectively reduces pain intensity in painful diabetic polyneuropathy, postherpetic neuralgia and hyperaesthesia of the skin in trigeminal neuralgia [15].

The analgesic effect is probably a result of the absorption of the drug, and it is associated with a presynaptic decrease of noradrenaline release from endings of the sympathetic nervous system and with an increase in the release of endogenous opioids (enkephalins) [16].

#### 4.5.3. Cannabinoids

Cannabinoids administered locally may induce analgesia through actions on cannabinoid receptors, CB1 and CB2 [17]. Actions through CB1 receptors may evoke a beneficial effect against neuropathic pain. Actions through CB2 may evoke a beneficial effect against inflammatory pain [18]. Analgesic effects of cannabinoids may also be induced by an inhibition of calcitonin release and an inhibition of a nerve growth factor.

## 5. Conclusions

There has been a continuous increase in knowledge and understanding of the pathophysiology of pain and its treatment. There are several routes of analgesic administration: oral, sublingual, buccal, intranasal, inhaled, subcutaneous, intravenous, intramuscular, rectal, intramedullary, intrathecal, transdermal and topical. Although most patients suffering from pain are treated with analgesics administered by oral and parenteral routes, there is increasing scientific evidence based on well-documented clinical studies that locally administered drugs may be at least as effective as those administered by the oral route [19]. An advantage of the topical route of drug administration is that it has a much better profile for adverse effects because they are designed for local pain treatment with minimal systemic effects. This refers especially to those groups of drugs in which systemic absorption is negligible. Transdermal and topical opioid administration is also associated with lower risk of addiction compared to oral and parenteral routes of opioid analgesics administration.

It should be remembered that simple methods of pain treatment are available through the topical administration of some drugs [20]. Through the topical route, the following analgesics may be administered: opioids, NSAIDs, and drugs acting locally through receptors and/or ion channels i.e., 5% lidocaine, EMLA cream, 8% capsaicin, tricyclics, nitrates, α–2–agonists, and cannabinoids (Table 5). Opioids may be administered by a transdermal route, which offers non–invasive and convenient route of analgesics administration that is especially indicated for patients with stable pain syndromes and those with GI tract disturbances.

## Figures and Tables

**Table 1:** Transdermal application of opioid analgesics.

Drug	Mechanism of Action	The Form of the Drug	Indications
Transdermal fentanyl	Agonist of µ opioid receptors	Patches releasing 12.5, 25, 50, 75 and 100 µg/h changed every 72 h	Chronic cancer–related and non–malignant pain
Transdermal buprenorphine	Partial agonist of µ opioid receptors Agonist of δ and weak antagonist of κ opioid receptors Agonist of ORL–1 (Opioid Receptor Like 1) receptors	Patches releasing 5, 10, and 20 µg/h changed every 7 days Patches releasing 35, 52.5 and 70 µg/h changed every 72–96 h	Chronic non–malignant pain, especially neuropathic pain syndromes Chronic cancer–related pain, especially neuropathic pain syndromes

**Table 2:** Basic data of transdermal fentanyl patches.

Dose (µg/h)	Daily Dose (mg/24 h)	Amount of Drug in One Patch (mg)	Duration of Analgesic Action
12.5	0.3	2.1	72 h (3 days)
25	0.6	4.2	
50	1.2	8.4	
75	1.8	12.6	
100	2.4	16.8	

**Table 3:** Basic data of transdermal buprenorphine patches.

Dose (µg/h)	Daily Dose (mg/24 h)	Amount of Drug in One Patch (mg)	Period of Analgesic Action
5	0.1	5	168 h (7 days)
10	0.2	10	
20	0.4	20	
35	0.8	20	96 h (4 days)
52.5	1.2	30	
70	1.6	40	

**Table 4:** Non–steroidal anti-inflammatory drugs used for topical application and their usual concentrations.

Drug	Concentration
Ketoprofen	2.5%
Piroxicam	0.5%
Diclofenac	1–2%
Ibuprofen	5%
Indomethacin	1%
Etofenamate	5–10%
Felbinac	3%
Flufenamic acid	2.5–3%

**Table 5:** Topical application for locally acting drugs.

Drug	Mechanism of Action	The Form of the Drug	Indications
Morphine used topically	Agonist of µ opioid receptors on peripheral nerve endings. Morphine causes opening of channels for potassium ions, and subsequent neuronal hyperpolarisation, and closure of the calcium ions channels, which inhibits the release of pronociceptive neurotransmitters	0.1–0.2% water solution 0.1% gel	Pain associated with changes at oral mucosa evoked by radiotherapy and chemotherapy (rinsing mouth with morphine aqueous solution) Skin ulcerations, skin damage induced by radiotherapy and pressure sores
NSAIDS (Nonsteroidal Anti–inflammatory Drugs)	Mechanism of action of a topical NSAIDs is likely related to anti-inflammatory action through prostaglandin synthesis inhibition via its adenosine triphosphate sensitive K^+^ channel opening property	Emulsion Patch Gel	Muscle and skeletal pain, also after injuries. Soft tissue pain. Rheumatic diseases. Osteoarthritis
Local anesthetics: EMLA cream Patches containing 5% lidocain	Local anesthetic agents suppress the activity of peripheral sodium channels within sensory afferents and subsequent pain transmission and preferentially block hyperexcitable cells	EMLA cream is a lotion oil–water containing lidocaine and prilocaine in a ratio 1:1. Patches containing 5% lidocain	EMLA cream: Skin anesthesia alleviating acute pain in surgery limited to small depth of the skin (excision of minor skin changes, fixing intravenous line, painful biopsies). Localized peripheral neuropathic pain, for example postherpetic neuralgia (PHN). 5% lidocain patches A first line drug in the treatment of a localized, peripheral neuropathic pain (especially with accompanying allodynia), alone or in combination with another first line drug. Central pain induced by spinal cord compression caused by a metastatic tumor to subarachnoid space
Capsaicin	Capsaicin is an of agonist of TRPV1 (Transient Receptor Potential Vanilloid 1) receptor on Aδ and C fibers causes opening of channels for calcium ions, their sharp influx to cells and subsequent reversible damage of mitochondrion, and in long–term atrophy of peripheral skin nerves, it also causes elimination of neurotransmitter (substance P) from nerve fibres endings	Ointment of a low (below 1%) concentration Patch with 8% capsaicin	Ointment of a low (below 1%) concentration: syndrome of burning mouth, neuropathic face pain, trigeminal neuralgia, reflex sympathetic dystrophy, neuropathy in the course of HIV, osteoarthrosis, fibromyalgia, persisted postoperative pain (post–mastectomy pain syndrome) Patch with 8% capsaicin: PHN and neuropathy in the course of HIV
Tricyclic antidepressants	Tricyclic antidepressants activate a descending antinociceptive system through inhibition of reuptake of serotonin and noradrenalin from synaptic cleft, as well as induce blockade of NMDA and adenosine receptors and sodium ions channels.	Cream: 5% doxepin, 10% amitryptyline	Peripheral neuropathic pain: carpal tunnel syndrome, neuralgia of intercostal nerves postherpetic neuralgia, complex regional pain syndrome, painful diabetic neuropathy, meralgia paraesthetica, coccydynia, costochondritis, mucositis after chemotherapy, intractable itch.
Nitrates	Exogenous nitrates stimulate release of NO, which activate sensitive to ATP potassium channels and in this way induce analgesia in periphery	Patch	Rheumatoid arthritis, ankylosing spondylitis, osteochondritis, muscle–fascial pain, complex regional pain syndrome, bursitis, coccydynia, bone metastases, vulvodynia, venous leg ulcers
α–2–agonists (clonidine)	Clonidine is a presynaptic alpha-2-adrenergic receptor agonist and an agonist of imidazoline receptors. Activation of alpha-2 receptors leads to release of an inhibitory G-protein, which down-regulates adenylate cyclase and other second messengers responsible for initiating and maintaining the abnormal excitability of nociceptors. Activation of the I_2_-imidazoline subclass of receptors located on peripheral nerve endings may be responsible for additional mechanisms of analgesic activity of clonidine	0.1% gel or cream, transdermal patches	Painful diabetic neuropathy, migraine, face pain, complex regional pain syndrome

## References

[1] Mc Cleane G.T. (2008). Pain Management: Expanding the Pharmacological Options.

[2] Argof C.E. (2013). Topical Analgesics in the Management of Acute and Chronic Pain. Mayo Clin. Proc..

[3] Leppert W., Krajnik M., Wordliczek J. (2013). Delivery Systems of Opioid Analgesics for Pain Relief: A Review. Curr. Pharm. Des..

[4] Pergolizzi J.V., Mercadante S., Echaburu A.V., Van den Eynden B., Fragoso R.M., Mordarski S., Lybaert W., Beniak J., Orońska A., Slama O. (2009). The role of transdermal buprenorphine in the treatment of cancer pain: An expert panel consensus. Curr. Med. Res. Opin..

[5] Krajnik M., Zylicz Z., Finlay I., Luczak J., van Sorge A.A. (1999). Potential uses of topical opioids in palliative care—Report of 6 cases. Pain.

[6] Inui N., Kato D., Uchida S., Chida D., Takeuchi K., Kimura T., Watanabe H. (2012). Novel Patch for Transdermal Administration of Morphine. J. Pain Symptom Manag..

[7] Maniar K.H., Jones I.A., Gopalakrishna R., Vangsness C.T. (2017). Lowering side effects of NSAID usage in osteoarthritis: Recent attempts at minimizing dosage. Expert Opin. Pharmacother..

[8] Hans G., Sabatowski R., Binder A., Boesl I., Rogers P., Baron R. (2009). Efficacy and tolerability of 5% lidocaine medicated plaster for the topical treatment of post–herpetic neuralgia: Results of long-term study. Curr. Med. Res. Opin..

[9] Price R.C., Gandhi W., Nadeau C., Tarnavskiy R., Qu A., Fahey E., Stone L., Schweinhardt P. (2018). Characterization of a novel capsaicin/heat ongoing pain model. Eur. J. Pain.

[10] Baron R., Treede R.D., Birklein F., Cegla T., Freynhagen R., Heskamp M.L., Kern K.U., Maier C., Rolke R., Seddigh S. (2017). Tretment of painful radiculopathies with capsaicin 8% cutaneous patch. Curr. Med. Res. Opin..

[11] Knezevic N.N., Tverdohleb T., Nikibin F., Knezevic I., Candido K.D. (2017). Management of chronic neuropathic pain with single and compounded topical analgesics. Pain Manag..

[12] Finnerup N.B., Attal N., Haroutounian S., McNicol E., Baron R., Dworkin R.H., Gilron I., Haanpää M., Hansson P., Jensen T.S. (2015). Pharmacotherapy for neuropathic pain in adults: A systematic review and meta-analysis. Lancet Neurol..

[13] Sandig A.G., Compmany A.C., Campos F.F., Villena M.J., Naveros B.C. (2013). Transdermal delivery of imipramine and doxepin from newly oil-in-water nanoemulsion for an analgesic and anti-allodynic activity: Development, characterization and in vivo evaluation. Colloids Surf. B Biointerfaces.

[14] Lauretti G.R., Perez M.V., Reis M.P. (2002). Double-blind evaluation of transdermal nitroglycerine as an adjuvant to oral morphine for cancer pain management. J. Clin. Anesth..

[15] Campbell C.M., Kipnes M.S., Stouch B.C., Brady K.L., Kelly M., Schmidt W.K., Petersen K.L., Rowbotham M.C., Campbell J.N. (2012). Randomized control trial of topical clonidine for treatment of painful diabetic neuropathy. Pain.

[16] Wrzosek A., Woron J., Dobrogowski J., Jakowicka-Wordliczek J., Wordliczek J. (2015). Topical clonidine for neuropathic pain. Cochrane Database Syst. Rev..

[17] Pergolizzi J.V., Lequang J.A., Taylor R., Raffa R.B., Colucci D., NEMA Research Group (2018). The role of cannabinoids in pain control: The good, the bad, and the ugly. Minerva Anestesiol..

[18] Lötsch J., Weyer-Menkhoff I., Tegeder I. (2017). Current evidence of cannabinoid–based analgesia obtained in preclinical and human experimental settings. Eur. J. Pain.

[19] Pickering G., Martin E., Tiberghien F., Delorme C., Mick G. (2017). Localized neuropathic pain: An expert consensus on local treatments. Drug Des. Dev. Ther..

[20] Malec-Milewska M., Dobrogowski J., Wordliczek J., Woron J. (2014). Topical administration of drugs in pain treatment. Pharmacotherapy of Pain.

